# Booth, A., Brown, S. L., Landale, N. S., Manning, W. D., McHale, S. M. (eds): Early Adulthood in a Family Context: Springer, New York, 2012

**DOI:** 10.1007/s10680-012-9275-0

**Published:** 2012-08-28

**Authors:** Monika Mynarska

**Affiliations:** Institute of Psychology, Cardinal Stefan Wyszyński University, Wóycickiego 1/3, Warsaw, Poland

Family formation is increasingly delayed in the contemporary developed world. A few decades ago, young adulthood (age 18–24) was a period of family formation. Nowadays, the usual steps of entry into adulthood—marriage, childbearing—come much later in the life-course. The age 18–24 is no longer a time of making commitments. In 2000, the psychologist Jeffrey Arnett used the term “emerging adulthood” to describe this life stage, emphasizing its new nature. The aim of the edited volume “Early adulthood in a family context” is to improve our knowledge about it.

The book is based on papers presented at the 18th Annual Symposium on Family Issues in October 2011. It has an interesting construction. The five parts of the book represent the five thematic sessions at the symposium. As for the first four parts of the volume, each of them consists of one leading chapter and a few shorter ones. While the leading chapter is always the most extensive, the shorter contributions complement it with additional analyses, overviews of research findings, or methodological comments. As a result, each part of the book is a coherent piece and with each chapter the reader gets a wider or more detailed picture of the discussed topic. It is like witnessing an experts’ discussion in given field.

The first part, “The contemporary context of young adulthood”, and especially the first chapter by Settersten Jr., set the stage for the whole book. Settersten describes key demographic shifts in the transition to adulthood in contemporary USA and discusses the challenges posed by them to young people and their families of origin. He also comments on what can make youth’s transition to adulthood easier: individual skills and capacities, family support as well as institutional context. The other authors take it from there.

The remaining chapters in the first section bring more insights on the challenges of “emerging adulthood” and on how successful transition to adulthood can be facilitated. The second part of the volume, with the leading paper by Fingerman and colleagues, continues on this topic by looking at the role of parent–child relationships. The authors in this section present a rich picture of relationships between young adults and their parents and document patterns of parental support—material, practical, and emotional. They also look at how this support is important for entry into adulthood by analyzing the long-term consequences on children’s wellbeing and achievements in later life.

The third part of the book (built around the chapter by Giordano and colleagues) addresses the issues of romantic and sexual relationships in young adulthood. If young people form their families later, do they also enter stable relationships later? The authors present how the nature of romantic relationships changes from adolescence to young adulthood. Sexual encounters of young people are also explored.

Finally, the fourth section investigates experiences of those who become parents during young adulthood. As the leading chapter in this section (by Edin and Tach) shows, in the USA “most of the young adults who become parents do so in the context of a non-marital relationship”. Therefore, this section touches on the topics of young fragile families (unmarried parents) that often struggle with economic hardship.

The last, fifth section, consists of two summarizing chapters. The first one (by Arnett) provides a neat summary of why it is important to study “emerging adulthood”. The second (by Hardie and Stanik) is an extended review of what the book teaches us on the role of family context in early adulthood.

Geographically, all the researches presented in the volume are based in the USA. But even though some aspects of the discussed issues are country specific (e.g., the role of community colleges in facilitating the transition to adulthood), the issues themselves are definitely universal. A European reader will easily be able to see many parallels with the youth situation in his or her home country. Of course, we may ask or even doubt, whether the “emerging adulthood” is the same in USA and Europe. But would not that be a very interesting research question to address in future research?

The authors use a variety of datasets and research methods to paint a comprehensive picture of “emerging adulthood” in contemporary USA. The book offers valuable insight into how adolescents become adults and into how family is crucial in this process. It is less about demographic rates and more about describing challenges that young people face and support they receive (or expect to receive). So why is it important for demographers? If we understand how young people experience entry into adulthood, we will understand their choices in later life much better. Therefore, studies on “emerging adulthood” may inform and enlighten our thinking not only about youth transition to adulthood, but also about fertility and family dynamics.

